# Negative correlations can play a positive role in disordered quantum walks

**DOI:** 10.1038/s41598-021-84073-4

**Published:** 2021-02-25

**Authors:** Marcelo A. Pires, Sílvio M. Duarte Queirós

**Affiliations:** 1 Centro Brasileiro de Pesquisas Físicas, Rua Dr Xavier Sigaud, 150, Rio de Janeiro, RJ 22290-180 Brazil; 2 Associate to the National Institute of Science and Technology for Complex Systems, Rio de Janeiro, Brazil

**Keywords:** Information theory and computation, Quantum physics, Optics and photonics, Physics

## Abstract

We investigate the emerging properties of quantum walks with temporal disorder engineered from a binary Markov chain with tailored correlation, *C*, and disorder strength, *r*. We show that when the disorder is weak—$r \ll 1$—the introduction of negative correlation leads to a counter-intuitive higher production of spin-lattice entanglement entropy, $S_e$, than the setting with positive correlation, that is $S_e(-|C|)>S_e(|C|)$. These results show that negatively correlated disorder plays a more important role in quantum entanglement than it has been assumed in the literature.

## Introduction

Inasmuch as the random walk has been at the cradle of the development of processes and techniques through out one hundred-off years, the introduction of its quantum counterpart, the quantum walk^1^(QW), urged a range of prospective applications, namely those related to the Feynman’s quantum computer proposal made some 10 years earlier^2^. Formally defined by a succession of local and unitary operations on qubits, QWs have definitely established as the direct path to understand complex quantum phenomena by means of relatively simple protocols^3–5^ that can be reproduced in a laboratory^6–8^ or the development of quantum algorithms^5^. Explicitly, the quantum walk evolves on a Hilbert space, ${\mathscr {H}}_2\otimes {\mathscr {H}}_{\mathbb {Z}}$, by means of the combined application of two unitary operators , the operator ${\hat{R}}$ acts on subspace ${\mathscr {H}}_2$ and plays the role of quantum coin related to internal (spin) states, *s*, whereas the external states related to the subspace ${\mathscr {H}}_{\mathbb {Z}}$ change due to the *shift operator*, ${\hat{T}}$. The successive application of a time-evolution operator allows obtaining the probability of finding the walker at position *x* at time *t*, $P_t(x)$, is given by $P_t(x)= \sum _s \left| \Psi ^{\left( s\right) } _t \left( x \right) \right| ^2$, from which the statistical characterization of the QW is made. Along the years, the original model^1^ has given raise to multitude of variants, e.g., by changing the dimensionality of the walk, the topology of the lattice as well as disorder in the jump size and the angle defining the quantum coin^9–19^.

A comparison between the classical and the quantum walker protocol shows that in latter, the stochastic part is replaced by operations on an internal degree of freedom the walker—traditionally its spin. Given that the position state of the walker has to do with that internal state, it is therefore natural to ask how much both states relate. The most straightforward way to quantum mechanically assess the degree of relation between both states is to look at quantum entanglement, specifically the entanglement entropy. The study of entanglement is of crucial importance for quantum information theory either for fundamental or applied issues^20^. In this work, we analyse the impact in the quantum entanglement of the correlation function—a classical measure—defining the disorder introduced in the system by means of a Markov process for the coin operator angle. Those protocols allow experimental implementation with the state-of-art photonic platforms^21^. Especially, we focus on the relevance of negative correlations to enhancing or undermining entanglement.

As we will show, the arrangement of random disorder with negative correlation leads to the appearance of distinctive configurations that produce more spin-lattice entanglement than the corresponding case with positive correlation.

## Literature review

Carrying out computational simulations, the issue of entanglement between the internal and external degrees of freedom of a quantum walker was first studied in 2005^22^; there, it was conveyed the asymptotic coin-position entanglement entropy $S_{\mathrm{e}} \rightarrow 0.872\ldots$. Afterwards, that result was analytically corroborated^23^ and verified in linear-optical experiments^24^.

Those works only considered disorder-free configurations in which the coin and translation operator are homogeneous in space and time. However, as mentioned in the previous Section, the assumption of disorder in such operators has opened the door for novel phenomenologies. For instance, nongaussian distributions can emerge^25^ or unusual gaussian with hyperballistic spreading can take place^16^. In 2012, it was reported an enhancement of entanglement with random disorder^26^, a result that was analytically proven afterwards^15^. Subsequently, the entanglement analysis was extended for other protocols of disorder either in the coin operator^14,15,21,26–29,29–34^ or in the step operator^18,35,36^. Recently, it was brought forth the first model with disorder in the translation operator that displays both the strengthening of entanglement and tunable spreading from slower-than-ballistic to faster-than ballistic^17^ and even can be found within a relativistic context^37^. From an experimental perspective, using photonic platforms, it was possible to confirm non-static disorder can favor entanglement^21^.

QWs with correlated disorder were previously studied in both discrete-^38–40^ and continuous-time^41,42^ representations. In Refs.^39,41^, it was shown that randomness leads to a diffusive-like behavior even when correlation is present. Regardless, the question of how the coin-space entanglement is affected by the correlation in the disorder remains uncovered to the best of our knowledge.

**Figure 1:**
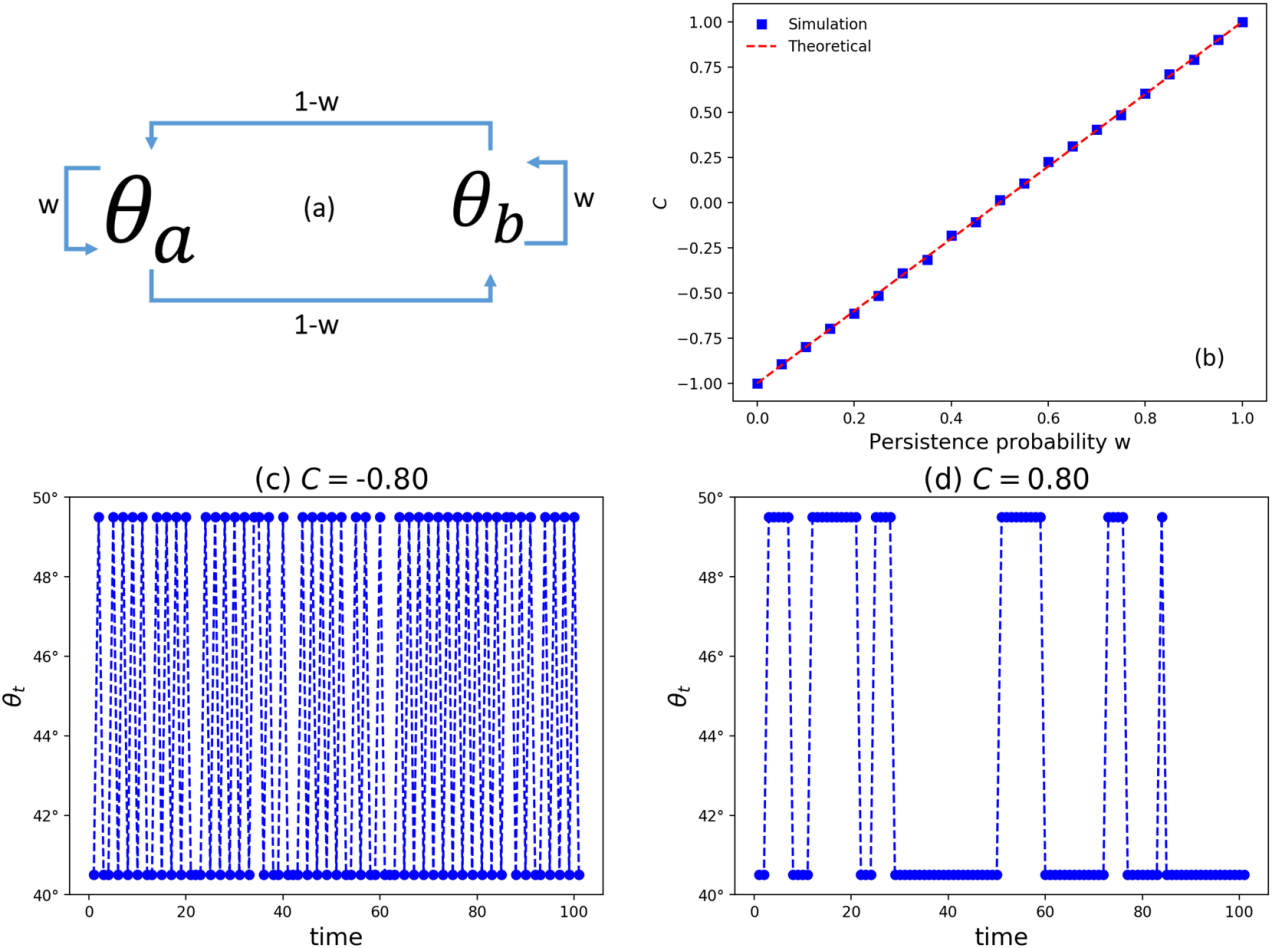
(**a**) Markov chain employed for tailoring the disorder with prescribed persistence probability *w* that provides the control of the autocorrelation with Eq. (29). (**b**) Autocorrelation of the sequence $\{\theta _t\}_{t=0}^{T}$. Monte Carlo simulations performed until $t_{max}=5000$. The theoretical line comes from Eq. (29). (**c**,**d**) Time series for $\theta _t$ engineered from Markov chains with $\theta _a=(1+r)\pi /4$ and $\theta _b=(1-r)\pi /4$ for $r=0.1$ and $C=\pm 0.8$ that comes from $w=\{0.1,0.9\}$ .

## Results

First, in Fig. 1 we present the time dependent properties in the angle of the coin operator, $\theta _t$. For details see “Methods”.

We start the characterization of our dynamics by computing the scaling exponent $\alpha$ of $m_2(t)\sim t^\alpha$, the second statistical moment of the probability distribution $P_t(x)$. With $\alpha$, we classify this process in terms of its diffusion features. Explicitly,

1 $$\begin{aligned} \alpha = \lim _{t \rightarrow \infty } \frac{\log m_2(t)}{\log t} \end{aligned}$$

2 $$\begin{aligned} m_2(t) = \overline{x^2}_t =\sum _x x^2P_t(x) . \end{aligned}$$

3 $$\begin{aligned} P_t(x) = |\psi _{t}^{D}(x)|^2 + |\psi _{t}^{U}(x)|^2 \end{aligned}$$

**Figure 2:**
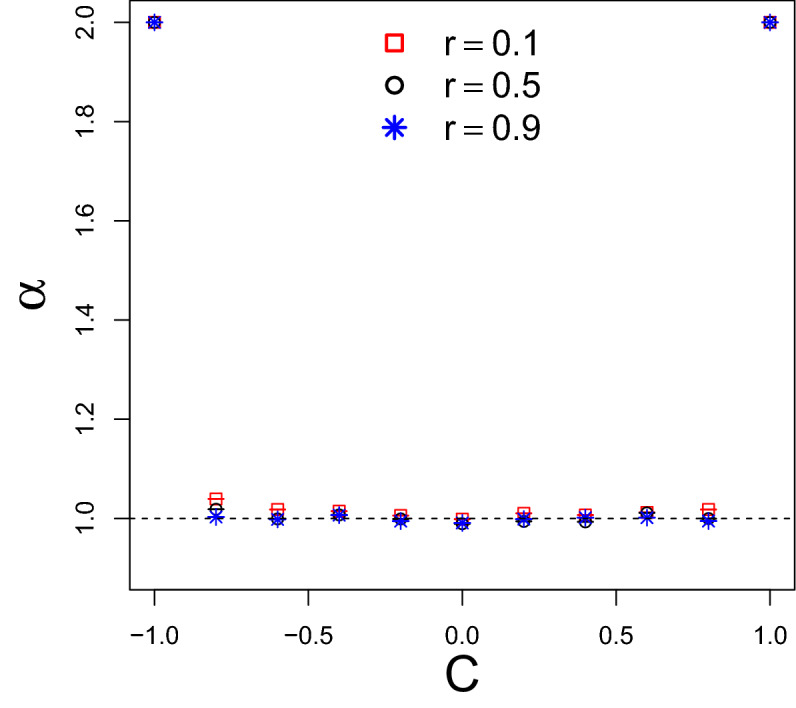
Diagram of the spreading regime versus the correlation *C* in the disorder for typical values $r=\{01,0.5,0.9\}$. We compute $\alpha$ from $m_2(t)\sim t^\alpha$ employing $t_{max}=5\times 10^5$ after discarding the transient $t<5\times 10^4$.

**Figure 3:**
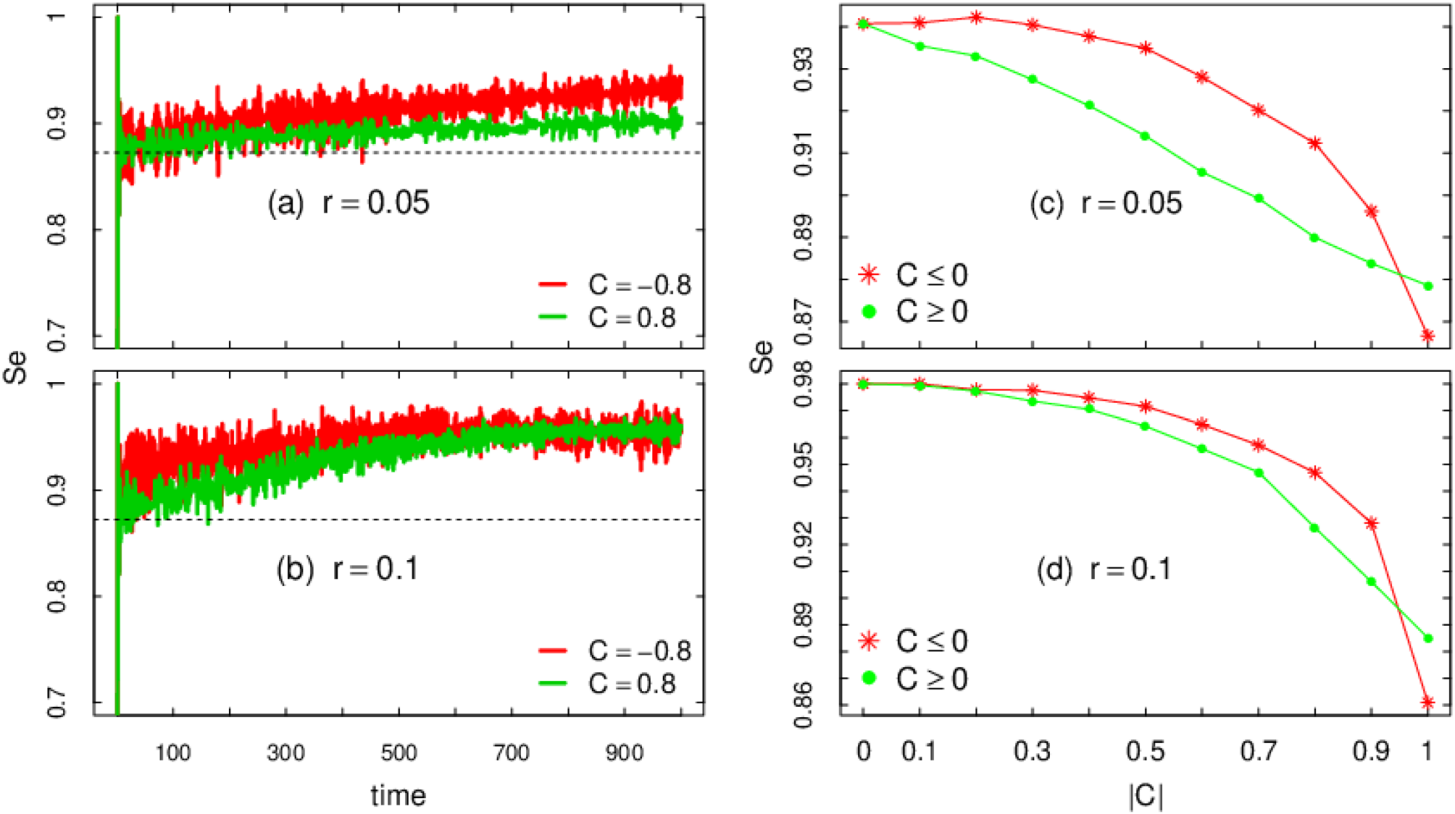
Von Neumann entanglement entropy $S_e$ versus (**a**,**b**) time, (**c**,**d**) *C*. Other parameters displayed in the panels. For (**c**,**d**) $S_e$ is computed at $t=500$ and each point is an average over 100 samples.

In Fig. 2, we see how $\alpha$ depends on the correlation in the disorder *C*. Let us first consider the particular cases previously addressed in the literature. Heed that we are using the variance so that $\alpha$ must be half should one is comparing the present results with diffusion defined by the standard deviation as made by some authors. As expected^43–46^ for uncorrelated disorder $C=0$, the diffusive-like behavior $\alpha = 1$ is achieved within error margin. In the maximal correlated situation, $C=1$, $\theta _t$ is constant, which explains the ballistic spreading $\alpha =2$. In the maximal anticorrelated case, $C=- 1$, $\theta _t$ is periodically alternating^47,48^ and this deterministic pattern leads to ballistic spreading as the standard quantum walk. For the other configurations, we observe that randomness induces a diffusive-like scaling exponent $\alpha$ for any correlation $|C|<1$. Even though this is not straightforward to interpret for a strong correlation $0.7<|C|<1$ this result is in agreement with Refs.^39,41^. That is to say, that randomness plays an important role in the path towards diffusive behavior in QWs with time-dependent coins, but spatial translational invariance, as done here. Event though randomness is an important ingredient for the emergence of diffusive scaling in quantum walks, we emphasize that it is not the only path to diffusion in QWs. For instance, two counterexamples are presented in Refs.^49,50^ where it was reported that some specific time dependence in $\theta _t$ can lead to diffusive-like spreading without any randomness.

The qubit-lattice entanglement is an important quantity that can also be quantified experimentally. To assess such feature we compute the Von Neumann entropy

4 $$\begin{aligned} S_e = - \mathrm {Tr} \left[ \rho ^c\log \rho ^c \right] , \end{aligned}$$

where $\rho ^c$ is the reduced density matrix obtained after tracing out the position degree of freedom

5 $$\begin{aligned} \rho ^c = \mathrm {Tr}_x(\rho ) \end{aligned}$$

and where $\rho$ is the full density matrix of the complete system

6 $$\begin{aligned} \rho = | \Psi \rangle \langle \Psi | \end{aligned}$$

Following^14,23,29^ we can obtain $S_e$ with expressions that are computationally faster to process

7 $$\begin{aligned} S_{\mathrm{e}} = - \sum _{\lambda \in \{\lambda ^{\pm }\}} \lambda \log _2 \lambda \end{aligned}$$

where the eigenvalues $\lambda ^{\pm }$ of $\rho ^c$ are

8 $$\begin{aligned} \lambda ^{\pm } = \frac{1}{2} \pm \sqrt{\frac{1}{4}-G_uG_d + |G_{ud}|^2 } , \end{aligned}$$

with

9 $$\begin{aligned} G_u = \sum _x |\psi _{t}^{U} (x)|^2 \end{aligned}$$

10 $$\begin{aligned} G_d = \sum _x |\psi _{t}^{D} (x)|^2 = 1 - G_u \end{aligned}$$

11 $$\begin{aligned} G_{ud} = \sum _x\psi _{t}^{U}(x) \left( \psi _{t}^{D}(x) \right) ^{*}. \end{aligned}$$

In Fig. 3a,b, we quantify the the entanglement between the internal and external degrees of freedom during the time evolution of the bipartite system. As initial condition, the QW starts from a state with minimum entropy $S_{\mathrm{e}}=0$, i.e., a separable state. For systems without disorder (dashed horizontal line in Fig. 3a,b), the asymptotic entanglement entropy is $S_{\mathrm{e}} \rightarrow 0.872\ldots$ as obtained in Refs.^22,23^. That value is quickly overcome for correlated arrangements in $\theta _t$ with $C=\pm 0.8$. Asymptotically, both cases lead to the maximum value, which is quite unexpected bearing in mind the strong correlation we are dealing with. The mathematical proof presented in^15^ requires randomness in $\theta _t$ for maximum $S_e$ as $t \rightarrow \infty$. In the present case, our results show that randomness in the presence of correlation also leads to $S_e \rightarrow 1$.

**Figure 4:**
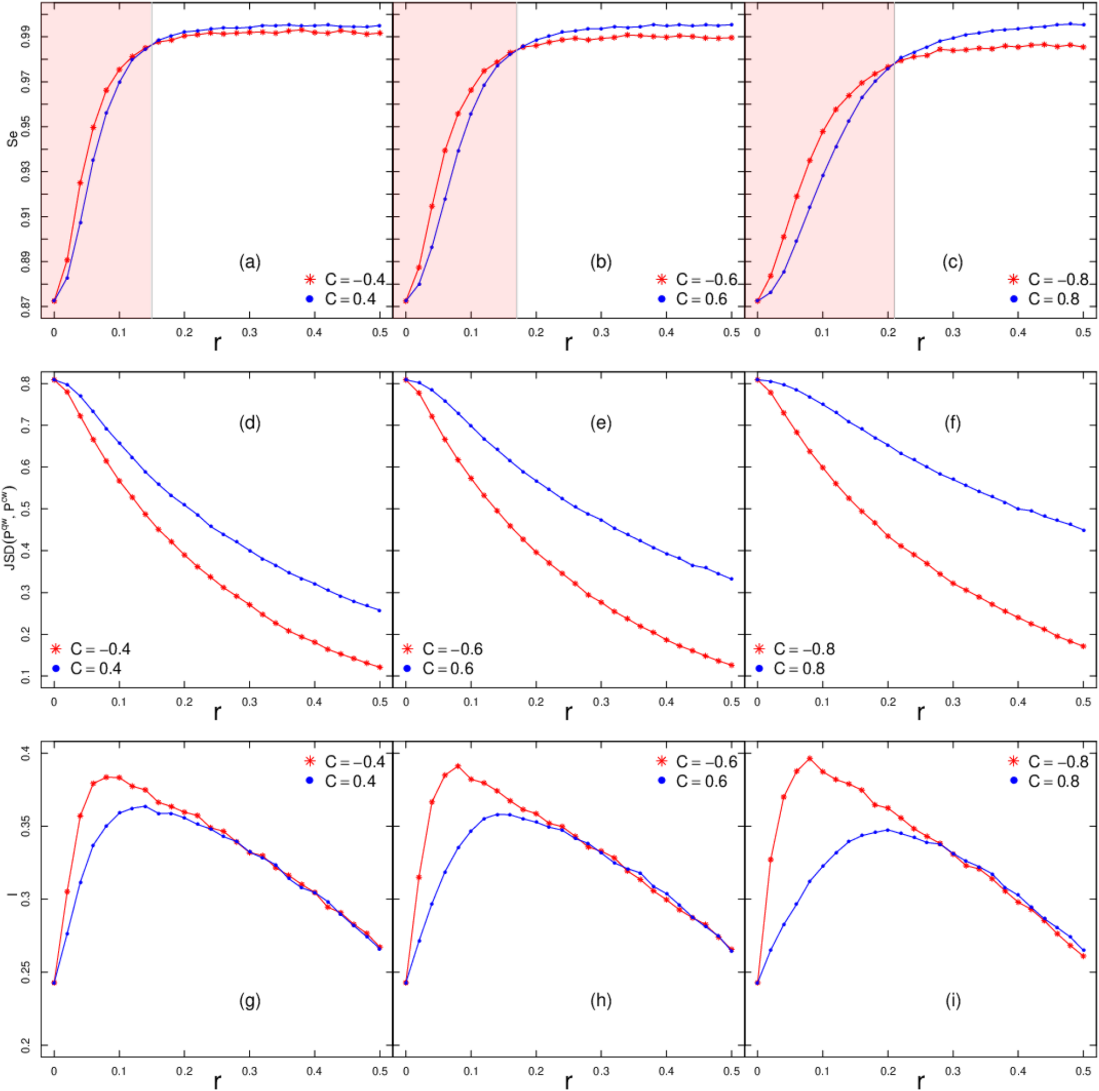
Entanglement entropy $S_e$ (**a**–**c**), Jensen–Shannon dissimilarity JSD (**d**–**f**), Interference measure *I* (**g**–**i**) at $t=500$ for increasing disorder strength *r* and $C=\{\pm 0.4,\pm 0.6, \pm 0.8\}$. Each point comes from an average over 500 samples. The red shadow area for (**a**–**c**) shows the regime where anticorrelation gives a clear advantage in terms of $S_e$.

Still in Fig. 3a,b, the case with negative correlation $C=-0.8$ produces more entanglement per unit of time than the case with $C=0.8$. This result persists if the strength of the temporal disorder increases from $r=0.0.5$ to $r=0.1$. Our findings open the way to the next question: how robust is such result for other correlation *C*? This question is addressed in Fig. 3c,d where we see that negative correlations are more prone to produce entanglement for any $0<|C|<1$. However, as the intensity of the disorder *r* increases this advantage peters out. This is further stressed by the results depicted in Fig. 4a–c.

**Figure 5:**
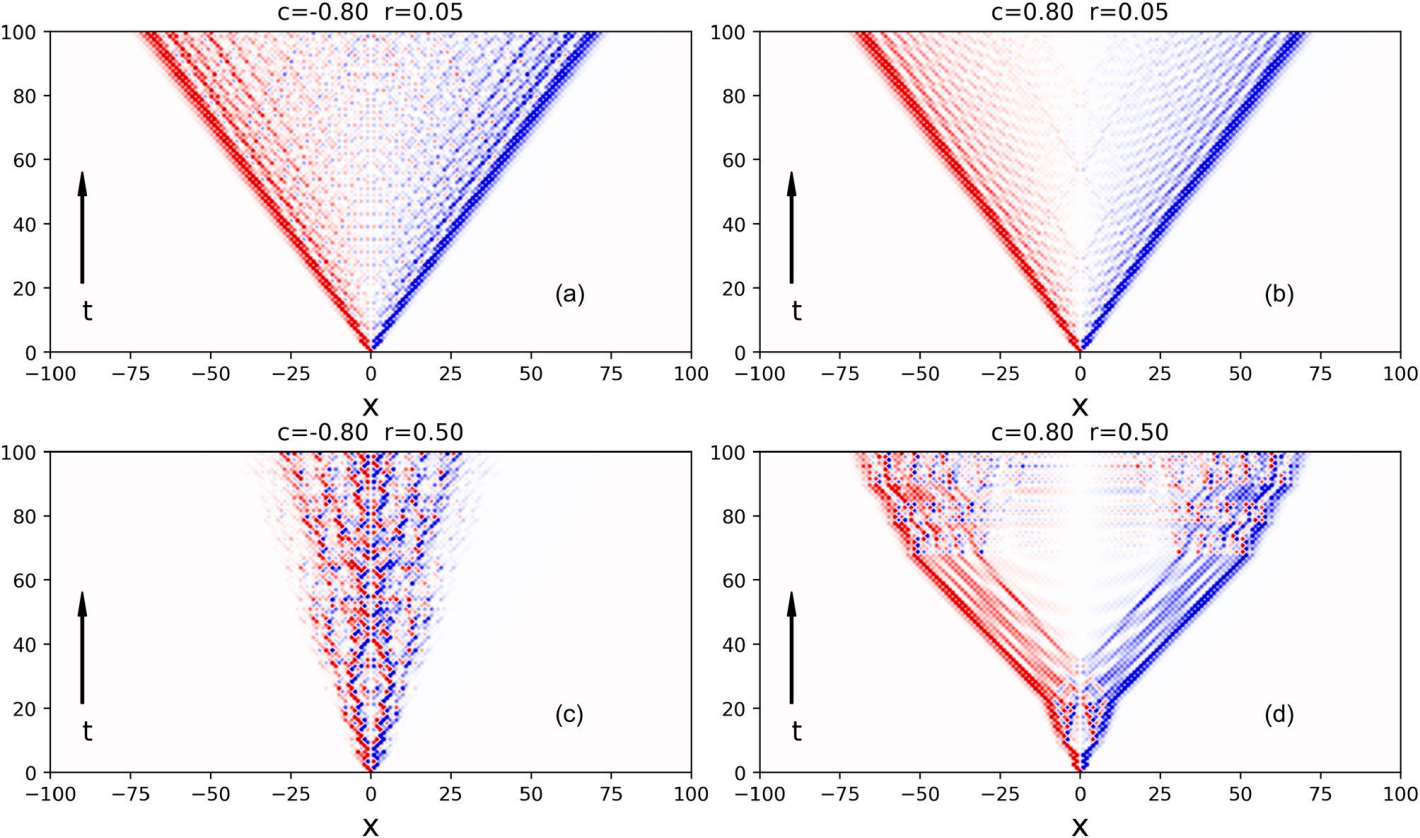
Spatiotemporal pattern for the normalized measure $A_t(x)/|A_t|^{\max }$ for $r=\{0.05,0.5\}$ and correlation $C=\pm 0.8$. Red profile: preponderance of $|\psi _{t}^{D}(x)|$. Blue profile: dominance of $|\psi _{t}^{U}(x)|$. In both cases, the darkness indicates the strength of $A_t(x)/|A_t|^{\max }$.

As aforementioned, we observe standard diffusion behavior, Fig. 2 typical of a classical system. Therefore, let us shed light on how much discrepancy is produced between the distributions arising from quantum and classical walks, $P^{\mathrm{qw}}_{t}(x)$ and $P^{\mathrm{cw}}_{t}(x)$. To that, we compute the Jensen-Shannon dissimilarity^51^.

12 $$\begin{aligned} JSD_t(P^{\mathrm{qw}},P^{\mathrm{cw}}) \equiv S(P^{mean}) -S_{mean}, \end{aligned}$$

where the first term is the entropy of the mean distribution^51^

13 $$\begin{aligned} P^{mean}(x) \equiv \frac{ P^{\mathrm{qw}}(x) + P^{\mathrm{cw}}(x) }{2}, \end{aligned}$$

and $S_{mean}$ is the mean entropy

14 $$\begin{aligned} S_{mean}= \frac{ S(P^{\mathrm{qw}}) + S(P^{\mathrm{cw}}) }{2}. \end{aligned}$$

An additional advantage of $JSD_t(P^{\mathrm{qw}},P^{\mathrm{cw}})$ refers to its property of being upper and lower bounded, $0\le JSD_t(P^{\mathrm{qw}},P^{\mathrm{cw}}) \le 1$.

In Fig. 4, we see how $JSD_t(P^{\mathrm{qw}},P^{\mathrm{cw}})$ changes with *r* for typical values of *C*. We generate the space-time probability distribution of the CW using the recursive relation,

15 $$\begin{aligned} P^{\mathrm{cw}}_{t+1}(x)=0.5P^{\mathrm{cw}}_{t}(x-1) + 0.5P^{\mathrm{cw}}_{t}(x+1). \end{aligned}$$

The disorder-free scenario, $r=0$, leads to the highest difference between $P^{\mathrm{qw}}_{t}(x)$ and $P^{\mathrm{cw}}_{t}(x)$. That dissimilarity decreases as the intensity of the disorder increases, because of increased overlap between the distributions arising from classical and quantum walk. In other words, the introduction of disorder with negatively correlated patterns in $\theta _t$ is more prone to disturb $P^{\mathrm{qw}}_{t}(x)$.

Besides $P_t(x)$, we can delve into the analysis of the relation between the components $\psi _{t}^{U}(x)$ and $\psi _{t}^{D}(x)$. Allowing for Refs.^25,52,53^, we compute $P_{t}^{U}(x)=|\psi _{t}^{U}(x)|^2$ and $P_{t}^{D}(x)=|\psi _{t}^{D}(x)|^2$ in terms of the coin parameter

16 $$\begin{aligned} P_{t+1}(x) = P_{t+1}^{U}(x) + P_{t+1}^{D}(x) = \cos ^2\theta _t \left( P_{t}^{U}(x+1) + P_{t}^{D}(x-1) \right) + \sin ^2\theta _t \left( P_{t}^{D}(x+1) + P_{t}^{U}(x-1) \right) + J_{t}(x) \end{aligned}$$

where the local interference term^53^ is

17 $$\begin{aligned} J_t(x)= & {} \sin 2\theta _t \mathfrak {R}\{ \psi _{t}^{U}(x-1) \psi _{t}^{D*}(x-1) -\psi _{t}^{U}(x+1) \psi _{t}^{D*}(x+1), \} \end{aligned}$$

where $\mathfrak {R}(z)$ stands for the real part of a complex number *z* and ${}^*$ its conjugate. The total interference over the chain at *t* is

18 $$\begin{aligned} I_t = \sum _x | J_t(x) | \end{aligned}$$

In Fig. 4, we see that differently from JSD $I_t$ displays a nonmonotonic behavior. More importantly, $I_t$ contains a signature of the regime where the negative correlation overcomes the positive correlation in terms of the entanglement production. Thus, the emergence of the regime where $S_e(-|C|)>S_e(|C|)$ comes at the expense of the emergence of a marked difference in the mutual modulation between the spin components at the level $\psi _{t}^{U}(x)$ and $\psi _{t}^{D}(x)$.

In order to better grasp the underlying mechanism behind our results let us inspect the local features of the spatial flux of probability over the chain^54^. This task can be achieved with

19 $$\begin{aligned} A_t(x) = |\psi _{t}^{U}(x)|^2 - |\psi _{t}^{D}(x)|^2 , \end{aligned}$$

In Fig. 5, we see that the embedded disorder in the coin operation leaves clear fingerprints in the spatiotemporal patterns in the normalized measure $A_t(x)/|A_t|^{\max }$ where $|A_t|^{\max } = \max _{x} A_t$ is the maximum over the chain updated for each *t*. For $C=-0.8$, the presence of pulse trains of short-time duration in $\theta _t$ induces a strengthening in the spatial interference of the components $|\psi _{t}^{U,D}(x)|^2$ which leads to a substantial flux of probability towards the central region. For $C=0.8$, the presence of pulse trains of long-time duration in $\theta _t$ stimulate a slowdown in the decay of the peaks near the edges. In both cases $C=\pm 0.8$, the increase in the magnitude of the disorder *r* leads to a increase in the evanescence of the fronts near the borders.

Finally, as the calculation of $S_e$ has the input the eigenvalues of $\rho ^c$, let us explore the asymptotic features of such reduced density matrix. To this task^15^, we pick the initial condition given by $\psi _0^U(x) = \psi _0^D(x) = \delta _{x,0}/\sqrt{2}$ (only for this analysis) and investigate the scaling behavior of the standard trace distance between states spanning consecutive time steps

20 $$\begin{aligned} D(t) \equiv \frac{1}{2} Tr\left( | \rho ^c(t) - \rho ^c(t-1) | \right) . \end{aligned}$$

**Figure 6:**
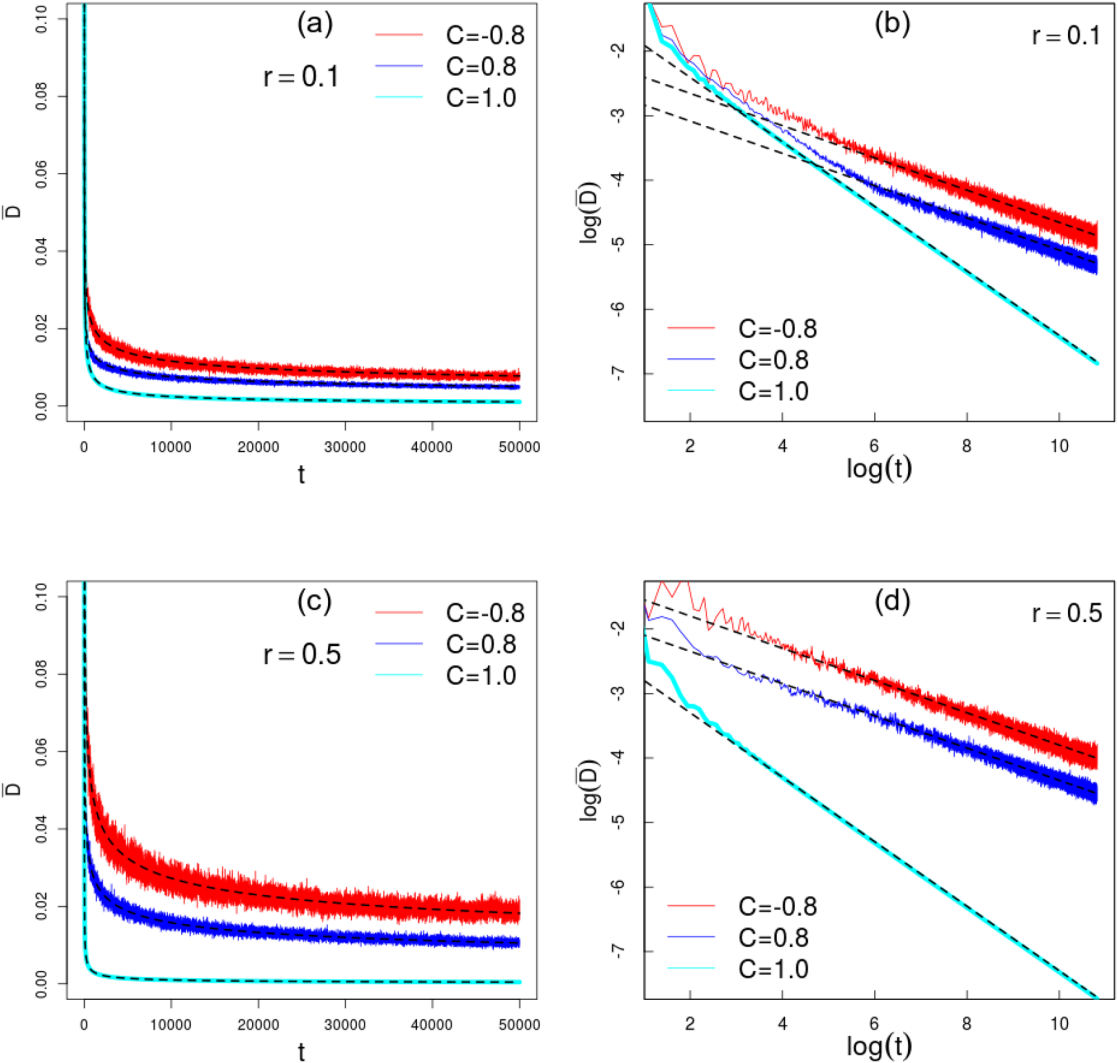
Time series for the average trace distance between states at adjacent instants. Outcomes for disorder strength $r=\{0.1,0.5\}$ as well as $C=\{-0.8,0.8, 1\}$. Full lines are obtained with 100 samples, except for the nonrandom QW with $C=1$. The superimposed dashed lines are the fits (values in Table 1) that are obtained after discarding 5 initials steps in order better describe the long-run tendency.

The numerical experiments in Fig. 6 and Table 1 highlight that average ${\bar{D}}(t)$ decays with a power-law in the long-run thus providing numerical evidence that $\rho ^c$ achieves an asymptotic limit ${\bar{D}}\rightarrow 0$ . It was shown^15^ the standard QW follows ${\bar{D}}\sim t^{-1/2}$ and the random QW follows ${\bar{D}}\sim t^{-1/4}$ for uncorrelated disorder. Our results show the algebraic decay ${\bar{D}}\sim t^{-1/4}$ holds even for temporal disorder with strong correlation $C=\{-0.8,0.8\}$. Such results – new to the literature on this subject to the best of our knowledge – provide novel insights about the time-dependence of the trace distance separating states at neighboring times.

## Discussion

Arenas where negative correlations can play a positive role, are rare. Recently, it was discovered a set of configurations in which energy transport at microscopic level can be heightened by negative correlations^13^. While in our scheme of disorder any value of correlation, $c \equiv |C|<1$, reduces the spreading to the diffusive regime, we present a framework where anti-correlated disorder confers a clear advantage for entanglement production in comparison with the corresponding protocol with positive correlation.

Entanglement in QWs has been addressed earlier with the introduction of modifications—relatively to the standard QW—either in the coin operator^14,15,21,26–29,29–34,55^ or in the step operator^17,18,35,36^. Nevertheless, the model we have introduced is able to combine the set of properties we obtained: (1) for weak disorder strength, $r<<1$, and assuming systems with either negative or positive correlation, $C = \mp c$, [eg, Fig. 1c,d where $c = 0.8$] the production of entanglement entropy (per timestep) is higher in the former than the latter; (2) asymptotically, randomness even in the presence of strong correlation also induces $S_e \rightarrow 1$. These results open up new possibilities for tailoring correlated disorder with target features, while keeping preserved $S_e \rightarrow 1$.

From an experimental point of view, the optical apparatuses in Ref.^56,57^ are promising candidates for implementing our proposal due to their flexibility in the design of the coin operator. Of particular interest here is the recent experiment conducted in^21^ where their compact photonic platform employs a time-dependent binary disorder in the coin operator that can be adapted to introduce our prescription of correlation. Their setup allows the reconstruction of the local spinor state for each site which in turn provides an indirect way to quantify the entanglement entropy and related measures. In a broader view, our work brings about further prospect in the definition of correlated disorder in experimental setups for realizing quantum walk^6–8^.

We reckon these results contribute to a better understanding of the relationship between correlated disorder and entanglement between the degrees of freedom of a quantum walk. On the one hand, Markov chains are very flexible and diverse with entire books devoted to its features. On the other hand, quantum walks are multigoal and versatile platforms. By bridging the two fields, our results strongly suggests that negatively correlated disorder could play a much more important role in applied issues^3–5,58^ as well as fundamental topics^59,60^.

**Table 1:** Fitted values used in Fig. 6.

–	$r=0.1$	$r=0.5$
$C=-0.8$	${\bar{D}}=(0.116056\pm 0.000050) t^{-1/4}$	${\bar{D}}=(0.272672\pm 0.000087) t^{-1/4}$
$C=0.8$	${\bar{D}}=(0.075485\pm 0.000053) t^{-1/4}$	${\bar{D}}=(0.157816\pm 0.000036) t^{-1/4}$
$C=1$	${\bar{D}}=(0.245038\pm 0.000051) t^{-1/2}$	${\bar{D}}=(0.100453\pm 0.000020) t^{-1/2}$

## Methods

### One-dimensional quantum walks

We consider a discrete-time evolution of a two-state quantum walk moving on a one-dimension lattice whose composite Hilbert space is ${\mathscr {H}}_2\otimes {\mathscr {H}}_{\mathbb {Z}}$. The composed state $|x\rangle \otimes |c\rangle =|x,c \rangle$ indicates the position $x \in {\mathbb {Z}}$ of a QW with internal degree of freedom (up/down), $c=\{U,D\}$. That degree of freedom, $c=\{U,D\}$, is associated with the corresponding space-time dependent amplitude of probability $\psi _t^{U,D}(x)$, respectively. At a given time $t \in {\mathbb {N}}$ we can write the full wave function $\Psi _t$ as

21 $$\begin{aligned} \Psi _t = \sum _{x \in {\mathbb {Z}}} \left( \psi _t^U(x) | U \rangle + \psi _t^D(x) | D \rangle \right) \otimes | x \rangle \end{aligned}$$

The time evolution of the QW is governed by

22 $$\begin{aligned}&\Psi _{t+1}={{\hat{W}}} \Psi _t \end{aligned}$$

23 $$\begin{aligned}&{{\hat{W}}} = {\hat{T}}({\hat{R}}\otimes \text {Id}_{\mathbb {Z}}) \end{aligned}$$

with the identity operator $\text {Id}_{\mathbb {Z}}=\sum _{x \in {\mathbb {Z}}} |x \rangle \langle x|$ and:

- The coin operator:
  24 $$\begin{aligned} {\widehat{R}}: {\left\{ \begin{array}{ll} |x, U \rangle \rightarrow \cos \theta _t |x, U \rangle + \sin \theta _t |x, D \rangle \\ |x, D \rangle \rightarrow \sin \theta _t |x, U \rangle -\cos \theta _t |x, D \rangle \end{array}\right. } \end{aligned}$$
  where the off-diagonal elements modulated by $\sin \theta _t$ are accountable for the coupling between the evolution of $\psi _t^U(x)$ and $\psi _t^D(x)$. The diagonal elements tempered by $\pm \cos \theta _t$ are responsible for propagation. The time-dependence of $\theta _t$ will be described in detail below.
- The state-dependent translation operator:
  25 $$\begin{aligned} {\widehat{T}}: {\left\{ \begin{array}{ll} |x, U \rangle \rightarrow |x+1, U \rangle \\ |x, D \rangle \rightarrow |x-1, D \rangle \end{array}\right. } \end{aligned}$$
  Meaning that the hopping-induced flux of probability takes place to neighbor sites.

Now we set the initial condition as

26 $$\begin{aligned} \psi _0^U(x) = \frac{1}{\sqrt{2}} \delta _{x,0} ,\quad \psi _0^D(x) = \frac{ i }{\sqrt{2}} \delta _{x,0} , \end{aligned}$$

### Tailoring disorder with Markov chains

At each time step *t*, we generate a random variable $z_t = \{a=-1,b=1\}$ following a two-state Markov chain with transition probability matrix:

27 $$\begin{aligned} M = \begin{pmatrix} p_{a \rightarrow a} &{} p_{a \rightarrow b} \\ p_{b \rightarrow a} &{} p_{b \rightarrow b} \end{pmatrix} \quad = \begin{pmatrix} w &{} 1-w \\ 1-w &{} w \end{pmatrix} \end{aligned}$$

where *w* is the persistence probability. Explicitly, *w* quantifies the probability of a given value to persist in the same state in the next step $t+1$. Thus, the switching probability is $1-w$. See Fig. 1a.

We set the baseline angle $\theta _0$, the kicking strength *r*. Then we define $\theta _a=(1+r)\theta _o$ and $\theta _b=(1-r)\theta _o$. This protocol is complete with the equation

28 $$\begin{aligned} \theta _t = \frac{(1-z_t)\theta _a}{2} + \frac{(1+z_t)\theta _b}{2} = {\left\{ \begin{array}{ll} \theta _a &{} \text { if } z_t=-1 \\ \theta _b &{} \text { if } z_t=1 \end{array}\right. } \end{aligned}$$

Explicitly, we start with $z_0=-1$ then with probability *w* we pick $z_1=z_0$, otherwise $z_1=-z_0$. This procedure is repeated iteratively until the desired time. With the time series $z_t$, we apply Eq. (28) to obtain the time series for $\theta _t$. With this routine, each kick train has a changeable length, but constant amplitude $(1 \pm r)\theta _o$. In all circumstances, we fixed $\theta _o=\pi /4$. As $0\le r\le 1$, then $\theta _a=(1+r)\pi /4\in [\pi /4,\pi /2]$ and $\theta _b=(1-r)\pi /4\in [0,\pi /4]$, then $0\le (1-r)\pi /4\le \theta _t\le (1+r)\pi /4\le \pi /2$.

For characterizing the similarity of the patterns through $\theta _t$ generated over time we employ the autocorrelation

29 $$\begin{aligned} C = \langle z_t z_{t-1} \rangle = \frac{1}{T-1} \sum _{t=1}^{T} z_t z_{t-1} = 2w - 1. \end{aligned}$$

In Fig. 1b, we show the estimated autocorrelation obtained from the Monte Carlo simulation of the disorder and using $C=\langle z_t z_{t-1} \rangle$.

Depending on the magnitude of the correlation we impose on the system The patterns in $\theta _t$ show different levels of similarity during the temporal evolution . The time series displayed in Fig. 1c,d illustrate the antipersistent patterns for $C=-0.8$ (marked by pulse trains of short duration) as well as the persistent patterns for $C=0.8$ (marked by pulse trains of long duration).

Apart from the transition matrix in Eq. (27), we generate a sequence, $\{\theta _t\}_{t=0}^{T}$, with the prescribed correlation *C*, which has the interesting property of being unbiased since the fractions of each ingredient in $\{\theta _t\}_{t=0}^{T}$ are

30 $$\begin{aligned} f_a= & {} \frac{p_{ab}}{p_{ba}+p_{ab}} = 50\% \quad t>>1, \end{aligned}$$

31 $$\begin{aligned} f_b= & {} \frac{p_{ba}}{p_{ba}+p_{ab}} = 50\% \quad t>>1, \end{aligned}$$

where $$p_{ba}=p_{ab}=1-w$$.

## References

[1] Aharonov Y, Davidovich L, Zagury N (1993). Quantum random walks. Phys. Rev. A.

[2] Feynman RP (1982). Simulating physics with computers. Int. J. Theor. Phys..

[3] Ambainis A (2003). Quantum walks and their algorithmic applications. Int. J. Quantum Inf..

[4] Venegas-Andraca SE (2008). Quantum walks for computer scientists. Synth. Lect. Quantum Comput..

[5] Portugal R (2013). Quantum Walks and Search Algorithms.

[6] Wang J, Manouchehri K (2013). Physical Implementation of Quantum Walks.

[7] Gräfe M, Szameit A (2016). Integrated photonic quantum walks. J. Phys. B Atom. Mol. Opt. Phys..

[8] Neves L, Puentes G (2018). Photonic discrete-time quantum walks and applications. Entropy.

[9] Ribeiro P, Milman P, Mosseri R (2004). Aperiodic quantum random walks. Phys. Rev. Lett..

[10] Bañuls MC, Navarrete C, Pérez A, Roldán E, Soriano JC (2006). Quantum walk with a time-dependent coin. Phys. Rev. A.

[11] Kendon VM (2006). A random walk approach to quantum algorithms. Philos. Trans. R. Soc. A.

[12] Attal S, Petruccione F, Sabot C, Sinayskiy I (2012). Open quantum random walks. J. Stat. Phys..

[13] Uchiyama C, Munro WJ, Nemoto K (2018). Environmental engineering for quantum energy transport. NPJ Quantum Inf..

[14] Zeng M, Yong EH (2017). Discrete-time quantum walk with phase disorder: Localization and entanglement entropy. Sci. Rep..

[15] Vieira R, Amorim EPM, Rigolin G (2013). Dynamically disordered quantum walk as a maximal entanglement generator. Phys. Rev. Lett..

[16] Di Molfetta G, Soares-Pinto DO, Queirós SMD (2018). Elephant quantum walk. Phys. Rev. A.

[17] Pires MA, Di Molfetta G, Queirós SMD (2019). Multiple transitions between normal and hyperballistic diffusion in quantum walks with time-dependent jumps. Sci. Rep..

[18] Pires MA, Queirós SMD (2020). Quantum walks with sequential aperiodic jumps. Phys. Rev. E.

[19] Vakulchyk I, Fistul MV, Flach S (2019). Wave packet spreading with disordered nonlinear discrete-time quantum walks. Phys. Rev. Lett..

[20] Horodecki R, Horodecki P, Horodecki M, Horodecki K (2009). Quantum entanglement. Rev. Mod. Phys..

[21] Wang Q-Q (2018). Dynamic-disorder-induced enhancement of entanglement in photonic quantum walks. Optica.

[22] Carneiro I (2005). Entanglement in coined quantum walks on regular graphs. New J. Phys..

[23] Abal G, Siri R, Romanelli A, Donangelo R (2006). Quantum walk on the line: Entanglement and nonlocal initial conditions. Phys. Rev. A.

[24] Su Q-P (2019). Experimental demonstration of quantum walks with initial superposition states. NPJ Quantum Inf..

[25] Shikano Y, Wada T, Horikawa J (2014). Discrete-time quantum walk with feed-forward quantum coin. Sci. Rep..

[26] Chandrashekar, C. Disorder induced localization and enhancement of entanglement in one-and two-dimensional quantum walks. arXiv:1212.5984 (arXiv preprint) (2012).

[27] Salimi S, Yosefjani R (2012). Asymptotic entanglement in 1d quantum walks with a time-dependent coined. Int. J. Mod. Phys. B.

[28] Rohde PP, Brennen GK, Gilchrist A (2013). Quantum walks with memory provided by recycled coins and a memory of the coin-flip history. Phys. Rev. A.

[29] Vieira R, Amorim EPM, Rigolin G (2014). Entangling power of disordered quantum walks. Phys. Rev. A.

[30] Di Molfetta G, Debbasch F (2016). Discrete-time quantum walks in random artificial gauge fields. Quantum Stud. Math.Found..

[31] Orthey AC, Amorim EP (2019). Weak disorder enhancing the production of entanglement in quantum walks. Braz. J. Phys..

[32] Singh S, Balu R, Laflamme R, Chandrashekar C (2019). Accelerated quantum walk, two-particle entanglement generation and localization. J. Phys. Commun..

[33] Montero M (2016). Classical-like behavior in quantum walks with inhomogeneous, time-dependent coin operators. Phys. Rev. A.

[34] Buarque ARC, Dias WS (2019). Aperiodic space-inhomogeneous quantum walks: Localization properties, energy spectra, and enhancement of entanglement. Phys. Rev. E.

[35] Sen P (2019). Scaling and crossover behaviour in a truncated long range quantum walk. Phys. A..

[36] Mukhopadhyay S, Sen P (2020). Persistent quantum walks: Dynamic phases and diverging timescales. Phys. Rev. Res..

[37] Arnault P (2020). Quantum simulation of quantum relativistic diffusion via quantum walks. J. Phys. A Math. Theor..

[38] Romanelli A, Siri R, Micenmacher V (2007). Sub-ballistic behavior in quantum systems with lévy noise. Phys. Rev. E.

[39] Ahlbrecht A, Vogts H, Werner AH, Werner RF (2011). Asymptotic evolution of quantum walks with random coin. J. Math. Phys..

[40] Mendes CVC, Almeida GMA, Lyra ML, de Moura FABF (2019). Localization-delocalization transition in discrete-time quantum walks with long-range correlated disorder. Phys. Rev. E.

[41] Yin Y, Katsanos DE, Evangelou SN (2008). Quantum walks on a random environment. Phys. Rev. A.

[42] Rossi MAC, Benedetti C, Borrelli M, Maniscalco S, Paris MGA (2017). Continuous-time quantum walks on spatially correlated noisy lattices. Phys. Rev. A.

[43] Wójcik DK, Dorfman JR (2003). Diffusive-ballistic crossover in 1d quantum walks. Phys. Rev. Lett..

[44] Košík J, Bužek V, Hillery M (2006). Quantum walks with random phase shifts. Phys. Rev. A.

[45] Joye A (2011). Random time-dependent quantum walks. Commun. Math. Phys..

[46] Ahlbrecht A (2012). Asymptotic behavior of quantum walks with spatio-temporal coin fluctuations. Quantum Inf. Process..

[47] Machida T, Konno N (2010). Limit theorem for a time-dependent coined quantum walk on the line. Nat. Comput..

[48] Rousseva J, Kovchegov Y (2017). On alternating quantum walks. Phys. A.

[49] Romanelli A (2009). Driving quantum-walk spreading with the coin operator. Phys. Rev. A.

[50] Panahiyan S, Fritzsche S (2018). Controlling quantum random walk with a step-dependent coin. New J. Phys..

[51] Lin J (1991). Divergence measures based on the shannon entropy. IEEE Trans. Inf. Theory.

[52] Romanelli A (2004). Quantum random walk on the line as a Markovian process. Phys. A.

[53] Singh, S. & Chandrashekar, C. Interference and correlated coherence in disordered and localized quantum walk. arXiv:1711.06217 (arXiv preprint) (2017).

[54] Souza A, Andrade R (2013). Coin state properties in quantum walks. Sci. Rep..

[55] Gratsea A, Lewenstein M, Dauphin A (2020). Generation of hybrid maximally entangled states in a one-dimensional quantum walk. Quantum Sci. Technol..

[56] Xue P (2015). Experimental quantum-walk revival with a time-dependent coin. Phys. Rev. Lett..

[57] Geraldi A (2019). Experimental investigation of superdiffusion via coherent disordered quantum walks. Phys. Rev. Lett..

[58] Montanaro A (2016). Quantum algorithms: An overview. NPJ Quantum Inf..

[59] Kitagawa T (2012). Topological phenomena in quantum walks: Elementary introduction to the physics of topological phases. Quantum Inf. Process..

[60] Wu J, Zhang W-W, Sanders BC (2019). Topological quantum walks: Theory and experiments. Front. Phys..

